# Primary healthcare worker knowledge related to prenatal and immediate newborn care: a cross sectional study in Masindi, Uganda

**DOI:** 10.1186/1472-6963-14-65

**Published:** 2014-02-11

**Authors:** Richard Mangwi Ayiasi, Bart Criel, Christopher Garimoi Orach, Elizabeth Nabiwemba, Patrick Kolsteren

**Affiliations:** 1 Department of Community Health and Behavioural Sciences, Makerere University School of Public Health, Mulago Hospital Complex, P.O Box 7072, Kampala, Uganda; 2 Institute of Tropical Medicine, Nationalestraat 155, Antwerp B-2000, Belgium

## Abstract

**Background:**

Global neonatal mortality remains unacceptably high. Health workers who attend to prenatal and postnatal mothers need to be knowledgeable in preventive and curative care for pregnant women and their newborn babies. This study aimed to determine the level of knowledge related to prenatal and immediate newborn care among primary healthcare workers in Masindi, Uganda.

**Methods:**

A cross-sectional study was conducted. Interviews comprised of 25 multiple-choice questions were administered to health workers who were deployed to offer prenatal and postnatal care in Masindi in November 2011. Questions were related to four domains of knowledge: prenatal care, immediate newborn care, management of neonatal infections and identifying and stabilizing Low-Birth Weight (LBW) babies. Corresponding composite variables were derived; level of knowledge among health workers dichotomized as ‘adequate’ or ‘inadequate’. The chi-square statistic test was used to examine associations with independent variables including level of training (nursing assistant, general nurse or midwife), level of care (hospital/health centre level IV or health centre level III/II) and years of service (five years or less, six years or more).

**Results:**

183 health workers were interviewed: general nurses (39.3%), midwives (21.9%) and nursing assistants (38.8%). Respectively, 53.6%, 46.5%, 7.1% and 56.3% were considered to have adequate knowledge in prenatal care, newborn care, management of neonatal infections and identifying/stabilizing LBW babies. Being a general nurse was significantly associated with having adequate knowledge in identifying and stabilizing LBW babies (*p* < 0.001) compared to being a nursing assistant. Level of care being hospital/health centre level IV was not significantly associated with having adequate knowledge in prenatal or newborn care with reference to health centres of level III/II.

**Conclusion:**

Knowledge regarding prenatal and newborn care among primary healthcare workers in Masindi was very low. The highest deficit of knowledge was in management of neonatal infections. Efforts are needed to orientate health workers regarding prenatal and newborn care especially the offer of infection management among newborns. Similar levels of knowledge between health workers deployed to hospital/health centre level IV and health centres of level III/II raise important implementation questions for the referral system which is crucial for maternal and newborn survival.

## Background

Worldwide, approximately four million newborns die every year before completing one month of life [1] jeopardizing the Millennium Development Goal (MDG) 4 target of reducing child mortality by two-thirds by the year 2015 [2]. It is widely acknowledged that MDG 4 target for child survival cannot be achieved without a particular focus on newborn health, especially during the first seven days of life [1,3]. Newborn complications resulting from hypothermia, infection and birth asphyxia that occur within the first seven days following birth contribute to the highest burden of morbidity and mortality [4]. Access to appropriate educational messages and treatment offered to women by health workers during the prenatal and immediate postnatal period is crucial in reducing related morbidity and mortality among newborn babies [5].

The global agenda for newborn health published in the Lancet Series [6] quantified the magnitude of the problem of neonatal mortality, outlined cost-effective interventions and suggested health system constraints that should be overcome [7]. The series concludes that success is possible without highly developed technology [7]. More recently the Global Newborn Action [8] advocates for acceleration and scale up of high-impact interventions to address major causes of newborn mortality [6]. It underscores the importance of key interventions and quality care for women and their babies and specifically calls for interventions days before, during and after birth. However, these interventions require deployment of health workers with adequate knowledge in maternal, child and newborn health [9]. The literature about human resources commonly describes the supply, choices of workplace, attraction, retention and attrition among health workers. Rarely is health worker knowledge on specific topics assessed yet appropriate implementation, among others, is dependent upon levels of knowledge [10]. In India, for example, high level of knowledge among Community Health Workers was considered pivotal for improving coverage and adherence to recommended newborn care practices [11]. In eastern Uganda, neonatal mortality autopsies demonstrated low levels of knowledge among health workers regarding prenatal and newborn care as a major cause of death [12]. Similar reasons have been highlighted in other disease conditions [13].

In Uganda, nearly all pregnant women make at least one antenatal care consultation with a health worker, 52% of deliveries take place at the health facility [14], and neonatal mortality remains relatively high at 29 per 1000 live births. Critically ill newborn babies often present for care at the formal health facilities where general nurses, midwifes and nursing assistants are routinely deployed [15]. These categories of health cadres inevitably form the first line of contact with prenatal, immediate postnatal women and newborn babies. It is therefore essential to establish their current levels of knowledge with regard to prenatal and newborn health [16].

This study aims to assess the level of knowledge related to prenatal and newborn care among primary health care workers providing ANC, immediate postnatal and newborn care in Masindi district, Uganda. The study further explores whether differences in knowledge are related to type of cadre, level of care or years of service after training. This study does not assess in-service training, notwithstanding its recognized importance in enhancing competence and performance of health workers [17,18]. This study is part of a larger enquiry that seeks to explore reasons for sub-optimal newborn care practices in Masindi.

## Methods

### Study site

This study was conducted in Masindi district in Western Uganda. Masindi district is located at 214 kilometres from the capital Kampala. It has a projected population of 603,000 inhabitants. The predominant cadre working at the health facilities are general nurses, nursing assistants and midwifes who are periodically redeployed across all health facilities in the district. In this region, about 97% of all pregnant women made at least one antenatal consultation, 42% made at least four antenatal consultations, and 43% delivered at a health facility (paper under review).

In Uganda, the health care system is organised into a four-tier system: hospital, health centres of levels IV, III and II. All levels of care are mandated to offer prenatal consultations and delivery services for pregnant women. Specifically, health centre of level II offers out-patients consultations. Health centre of level III offers outpatients, inpatients and laboratory services. Health centre of level IV and hospital offer caesarean operations and blood transfusion services in addition to outpatient and inpatient services. Hospitals serve as the main referral centre of the district health system. Masindi district has two referral general hospitals, one health centre of level IV, 10 health centres of levels III and 21 health centres of level II.

### Study population

Three categories of health workers (general nurses, midwives and nursing assistants) formed our primary target and were assessed for their levels of knowledge. The minimum entry level for pre-service training for general nurses and midwives is eleven years of education. The training curriculum of nursing cadres in the Ugandan health system is meant to produce polyvalent health workers capable of handling general nursing as well as maternal, child and newborn health. Midwives undergo a three-year training in which they are instructed on prenatal, postnatal and newborn care. They learn how to conduct normal deliveries, recognize danger signs and initiate timely referrals. General nurses receive also a three-year training mainly in bedside nursing but also aspects of midwifery which includes, the offer of comprehensive prenatal care, delivery care, immediate postnatal care and newborn care. Nursing assistants, on the other hand, have not undergone a formal training. They are health workers who have acquired nursing and midwifery skills by apprenticeship. Their basic level of education ranges from seven to eleven years. To date, nursing assistants in Uganda supply up to 50% of the human resources for health. General nurses, midwives and nursing assistants are routinely deployed in the different levels of care like health centres level II, III, IV or hospital and within these centres they can be assigned in different service points like maternity, children’s ward, out-patients department and so on. Given their number and contribution to the bulk of human resources for health, the government of Uganda, through its Ministry of Health decided to offer a three-month comprehensive training on general nursing and midwifery skills to all nursing assistants who were already enrolled in service.

### Sample size and selection

The sample size was estimated by using the formulae for cross-sectional studies [19]; assuming a health worker knowledge of 50%, with a sampling error of 8%. Considering a 10% non-response rate, 165 health workers needed to be recruited. All general nurses, midwives and nursing assistants deployed in health centres of level IV, III and II, general nurses working in the outpatients and children’s wards of the hospitals were eligible to participate in the study. Health workers currently deployed in surgical and medical wards of the hospitals were excluded since they don’t routinely offer consultation services for pregnant women or newborn babies. A list of all midwives, general nurses and nursing assistants was obtained from the district health office and stratified by the different category of cadres. The total sample was derived using computer generated random numbers following a ratio of midwives to general nurses and nursing assistant in the district 1:2:2. Sampling of the different cadres was done proportionate to their total numbers as follows: midwives (35), general nurses (70) and nursing assistants (70).

### Data collection

Interviews were conducted between November and December of 2011. Five research assistants were engaged and trained on the objectives of the study, the study tools and study methodology for two days. The research tool was adapted from Eriksson et. al [20] who used it in Vietnam to assess health worker knowledge regarding newborn care. Further modification of the tool was done based on the literature [21] (for tools refer to Additional file 1). Tools were pretested among health workers in the neighbouring district of Hoima. Research assistants visited one health centre at a time. At the health centre, informed consent was secured from health workers that were approached to participate in the study. Research assistants waited for each respondent to complete their questionnaire before engaging with the next respondent. Questionnaires were immediately retrieved by research assistants after they were completed. Questionnaires were administered to health workers who were found to be present on duty during the interview days. More than one visit was made to the health centre in case the sampled respondent was found to be absent. Telephone appointments were made for those who were out of their duty station, were on annual leave but resident within the district during the interview period. Twenty five multiple choice questions were administered by the trained research assistants. Interview questions were designed to assess knowledge on four broad areas of prenatal and newborn care: prenatal care, immediate newborn care, managing infections of the newborn and identifying and stabilizing LBW babies. Appropriate responses were coded as *yes = 1* while inappropriate responses were coded as *no = 0*.

### Dependent variables

Four composite variables were constructed from primary responses to measure levels of knowledge in four domains of prenatal and immediate postnatal care. *Prenatal care* (timing and frequency of ANC, routine ANC activities, routine observations during ANC, frequency of health education inclusive important messages offered, danger signs in pregnancy); *immediate newborn care* (initiation and frequency of breastfeeding, duration and cessation of breastfeeding, care for the cord during delivery, newborn resuscitation, postnatal assessment and timing); *managing infections* (newborn bleeding and vitamin K administration, managing eye infection, managing infected cord); *care for LBW babies* (identify and stabilize a LBW baby, care for LBW baby). For each of the themes, health workers were judged to have *‘adequate knowledge’* if they mentioned correctly any three of the prenatal care practices, any three of the five components of immediate newborn care, any two of the three options for managing infections and at least one of the two options for caring for LBW babies.

### Independent variables

Four independent variables were recorded. Whether the health worker was a nurse, midwife or nursing assistant (cadre of health worker); if health worker was currently deployed to - hospital, health centre levels IV, III or II (level of care); and finally, the number of years he/she has served after pre-service training (years in service).

### Data analysis

Data was entered in epiData computer software version 3.02. Data was cleaned and exported to STATA version 12 (College Station, Texas 77845 USA, 800-STATA). Frequency tables were generated. Associations between the level of knowledge in each of the four main themes were explored with independent variables, ‘*cadre of health worker’*, *‘level of care ‘and years of service*. As mentioned earlier, general nursing and midwifery training are meant to develop polyvalent health workers and therefore prepared to address routine problems like maternal and newborn care. Moreover, the Public Service Standing Orders for Uganda suggests regular deployment of health workers across different units and different levels of care [22]. The Standing Orders further suggest deployment of highly trained general nurses and midwives at higher levels of care (hospital and health centre level IV) [22]. Newly recruited health workers are expected to serve a two-year probation period and redeployment is foreseen after serving in a particular unit for a minimum of three years. Outpatients’ departments for health facilities serve as the first point of contact for all patients. Health workers deployed at the outpatients department conduct a triage before referral for further management to the relevant unit. Based on these arguments we considered important comparing the different levels of care. Put together, two years of probation and three years of first deployment, we considered a total of five years of initial service sufficient for a health worker to gain relevant experiences.

The chi-square statistic was used to examine for level of significance. The Bonferroni adjustment was applied to estimate levels of significance since multiple testing tends to increase the chances for finding significant variables [23,24]. In this analysis 12 repeated tests were done therefore the standard 0.05 level of significance was divided by twelve. A *p*-value equal or less than 0.004 was considered to be significant.

### Ethical approval

Ethical approval was obtained from the Institutional Review Board of the School of Public Health from Makerere University College of Health Sciences and the National Council of Science and Technology. Written consent was obtained from each participating health worker.

## Results

### Sample characteristics

We interviewed 183 health workers (Table 1): 72 general nurses (39.3%), 40 midwives (21.9%) and 71 nursing assistants (38.8%). They were either deployed in the maternity unit (66; 36.1%), children’s department (32; 17.5%) or working in the out patients department (85; 46.6%). Their years of service/experience after pre-service training ranged from 1 to 32 years; median 6 years [interquartile range (IQR): 3–9]. Respondents were predominantly females (94%). Other details are shown in Table 1.

**Table 1:** Characteristics of respondents

**Variable**	**Frequency n(%)**
**Cadre of health worker**	
Nursing assistant	71(38.8)
General Nurse	72(39.3)
Midwife	40(21.9)
**Years of experience**	
0 - 8 yrs	125(68.3)
9 - 16 yrs	44(24.0)
17 - 23 yrs	11(6.0)
24 - 32 yrs	3(1.7)
**Gender**	
Male	11(6.0)
Female	172(94.0)
**Deployment/Health facility**	
HC level II/III	111(60.7)
HC level IV/Hospital	72(39.3)
**Assignment**	
ANC/FP/ANC	66(36.1)
Other units	117(63.9)

### Knowledge on recommended prenatal care and newborn care practices

About 70% of health workers correctly mentioned the expected observations and important health education messages routinely offered during prenatal consultations. However, less than 40% could mention the correct timing for the first ANC visit, the optimal number of visits and basic interventions that are offered during prenatal consultations (Table 2). Overall, 98/183 (53.6%) were judged to have adequate knowledge about prenatal care (Table 3).

**Table 2:** Response to MCQ questionnaire to assess health worker knowledge

	**Variable**	**Frequency n(%)**
**1.**	**Timing of first ANC visit**	
	Amenorrhea of one month	57(31.2)
	Amenorrhea of two months	61(33.3)
	Amenorrhea of three months	63(34.4)
	I have no opinion	2(1.1)
**2.**	**Recommended number of ANC visits**	
	At least three visits	22(12.0)
	At least four visits	155(84.7)
	Any number of visits	6(3.3)
**3.**	Routine interventions during ANC*	
	History	164(89.6)
	Physical examination	168(91.8)
	Laboratory investigations	154(84.2)
	Health education	174(95.1)
	Assessment for referral	110(60.1)
**4.**	**Frequency of health education**	
	During every visit	179(97.8)
	Only once	4(2.2)
**5.**	Important discussions with mothers*	
	Danger signs in pregnancy	174(95.1)
	Birth preparation	169(92.4)
	Care for the newborn	142(77.6)
	Health facility delivery	172(94.0)
**6.**	Mentioned danger signs in pregnancy*	
	Swelling of face and feet	172(94.0)
	Excessive vomiting	166(90.7)
**7.**	Routine measurements during ANC*	
	Weight	172(94.0)
	BP	178(97.3)
	Height of funds	162(88.5)
**8.**	**Initiation of BF**	
	Within the first hour	158(86.4)
	1–6 hours	20(10.9)
	>6 hours	5(2.7)
**9.**	**Advise in case no Breast milk**	
	Give formula milk	32(17.5)
	Continue with BF even when milk is not coming	151(82.5)
**10.**	**Duration for exclusive BF**	
	One month	4(2.2)
	4 months	142(77.6)
	>6 Months	35(19.1)
	No opinion	2(1.1)
**11.**	**When to stop BF**	
	12 months	2(1.1)
	18 months	14(7.7)
	24 months	117(64.0)
	>2 years	46(25.1)
	No opinion	4(2.2)
**12.**	Newborn resuscitation*	
	Dry with cloth	111(60.7)
	Use ambu-bag	136(74.3)
	Suction of airway	160(87.4)
	Slap the baby	62(33.9)
	Pour cold water	17(9.3)
**13.**	**Prevention of newborn bleeding**	
	Breastfeed the child	9(4.9)
	Not necessary to give anything	15(8.2)
	Give vitamin K	63(34.4)
	Give vitamin K_1_	79(43.2)
	Have no opinion	17(9.30)
**14.**	**Doze of vitamin K**_**1**_	
	0.5 mg	91(49.7)
	1.0 gm	29(15.9)
	No opinion	63(34.4)
**15.**	**Treatment of eye infection**	
	Apply nothing	11(6.0)
	Apply breast milk in the eye	9(4.9)
	Clean eye with sterile water	122(66.7)
	Apply silver nitrate	31(16.9)
	No opinion	10(5.5)
**16.**	Care for the cord after delivery*	
	Clean hands	166(90.7)
	Clean instrument	163(89.1)
	Any sharp instrument	21(11.5)
**17.**	Care of the cord in case of infection*	
	Leave to dry	54(29.5)
	Wash with water and soap	89(47.0)
	Apply iodine	41(22.4)
	Apply antibiotic powder	40(21.9)
	Refer to hospital	152(83.1)
**18.**	Stabilizing the temperature of LBW baby*	
	Bath baby in water of appropriate temperature	72(39.3)
	Put on clothes and cover head	124(67.8)
	Skin-to-skin	132(72.1)
	Room temperature of 28–30 degrees Celsius	77(22.1)
	Near a radiator	15(8.2)
**19.**	**Definition of a LBW**	
	<3000 gms	9(4.9)
	<2500 gms	83(45.4)
	<1500 gms	38(20.8)
	<1000 gms	32(17.5)
	No opinion	21(11.5)
**20.**	Care for LBW baby*	
	Bath often	17(9.3)
	BF early and frequently	152(83.1)
	Keep the child warm	145(79.2)
	Prevent infection from developing	132(72.1)
**21.**	Importance of home visits*	
	To assess mother	149(81.4)
	To ask mother about baby	165(90.2)
	To assess baby for icterus	123(67.2)
**22.**	**The best timing for first postnatal visit**	
	Not important	1(0.6)
	During first three days	60(32.8)
	Between 3–7 days	92(50.3)
	Between day 8-14	23(12.6)
	I have no opinion	7(3.8)
**23.**	Who should conduct home visits*	
	VHT	102(55.7)
	Nurse	108(59.0)
	Midwife	171(93.4)

*Totals may not necessarily add up to 183 because of multiple responses in some instances; ANC-Antenatal care; BF-Breastfeeding; LBW-Low Birth-Weight; MCQ Multiple choice Question; VHT Village Health Team.

**Table 3:** Proportion of health workers with adequate knowledge in prenatal and newborn care

**Category**	**Variable**	**Proportion that made correct response n(%)**	**Proportion with adequate knowledge n(%)**
		**n = 183**	**n = 183**
Prenatal care	Timing & Frequency of ANC	51(27.9)	98(53.6)
	Routine ANC activities	73(39.9)	
	Routine Observations in ANC	157(85.8)	
	Frequency of Health Education & important messages	131(71.6)	
	Danger signs during pregnancy	116(63.4)	
Immediate Newborn care	Initiation of BF and Pre-lacteal feeds	140(76.5)	85(46.5)
	Duration and cessation of BF	94(51.4)	
	Care for the cord during delivery	132(72.1)	
	Newborn resuscitation	40(21.9)	
	Postnatal Timing & Assessment	36(19.7)	
Managing infection	Newborn bleeding and Vitamin K_1_	11(6.0)	13(7.1)
	Managing eye infection	31(16.9)	
	Managing infected cord	55(30.1)	
Care for LBW baby	Identify and stabilize a LBW	19(10.4)	103(56.3)
	Care for a LBW baby	93(50.8)	

ANC: antenatal care; LBW: Low birth weight.

Over 70% of health workers mentioned the correct time for initiation and duration for breastfeeding and appropriate care for the cord. Less than a quarter of them could correctly mention the optimal timing for the first postnatal care visit and newborn resuscitation (Table 2). Just about half 85/183 (46.5%) were judged to have adequate knowledge in immediate newborn care (Table 3).

Less than 30% of health workers could mention correctly management of a bleeding cord, infected eye or cord infection. About one in ten 10.4% (19/183) could correctly identify a LBW baby and suggest appropriate management (Table 3). Subsequently, 13/183 (7.1%) and 103/183 (56.3%) were judged to have adequate knowledge in infection management or caring for LBW babies, respectively (Table 3).

### Factors associated with level of knowledge

#### Level of training

In our preliminary assessments (Table 4), 50.7% (36/171) of nursing assistants, 51.4% (37/72) general nurses and 62.5% (25/40) of midwives were considered to have adequate knowledge in prenatal care. There was no statistical difference in the level of prenatal knowledge among general nurses (*p* = 0.232) and midwives (*p* = 0.935) with reference to nursing assistants (Table 4).

**Table 4:** Chi-Square statistics to test health worker knowledge regarding prenatal and newborn care

**Independent variable**		**Level of knowledge n(%)**		**OR[CI]**	***p*-value**
		**Adequate knowledge**	**Inadequate knowledge**		
		**Prenatal care (N = 183)**			
**Level of training**					
	Nurse Assistant	36(50.7)	35(49.3)	0	
	Nurse	37(51.4)	35(48.6)	1.02[0.53-1.98]	0.935
	Midwife	25(62.5)	15(37.5)	1.62[0.73-3.58]	0.232
**Level of care**					
	HC level III or II	59(53.2)	52(46.8)	0	
	Hospital or HC IV	39(54.2)	33(45.8)	1.04[0.57-1.89]	0.893
**Years of service**					
	0-5	39(49.4)	40(50.6)	0	
	6-32	59(56.7)	45(43.3)	1.34[0.75-2.43]	0.324
		**Immediate newborn care (N = 183)**			
**Level of training**					
	Nurse Assistant	27(38.0)	44(62.0)	0	
	Nurse	33(45.8)	39(54.2)	1.38[0.71-2.69]	0.345
	Midwife	25(62.5)	15(37.5)	2.72[1.22-6.04]	0.014
**Level of care**					
	HC level III/ II	49(44.1)	62(55.9)	0	
	Hospital/HC IV	36(50.0)	36(50.0)	1.27[0.70-2.29]	0.438
**Years of service**					
	0-5	36(45.6)	43(54.4)	0	
	6-32	49(47.1)	55(52.9)	1.06[0.59-1.92]	0.836
		**Managing infections on newborns (N = 183)**			
**Level of training**					
	Nurse Assistant	11(15.5)	60(84.5)	0	
	Nurse	8(11.1)	64(88.9)	0.68[0.26-1.81]	0.442
	Midwife	5(12.5)	35(87.5)	0.78[0.25-2.43]	0.667
**Level of care**					
	HC level III or II	14(12.6)	97(89.4)	0	
	Hospital or HC IV	10(13.9)	62(86.1)	1.12[0.47-2.67]	0.803
**Years of service**					
	0-5	8(10.3)	71(89.9)	0	
	6-32	5(4.8)	99(95.2)	0.45[0.14-1.44]	0.167
		**Identifying and stabilizing LBW Babies (N = 183)**			
**Level of training**					
	Nurse Assistant	27(38.0)	44(62.0)	0	
	Nurse	50(69.4)	22(30.6)	3.7[1.85-7.41]	0.000*
	Midwife	26(65.0)	14(35.0)	3.0[1.35-6.78]	0.007
**Level of care**					
	HC level III or II	60(54.1)	51(46.0)	0	
	Hospital or HC IV	43(59.7)	29(40.1)	1.20[0.69-2.30]	0.450
**Years of service**					
	0-5	44(55.7)	35(44.3)	0	
	6-32	59(56.7)	45(43.3)	1.04[0.58-1.88]	0.889

*p-value < 0.004.

Considering the level of knowledge for newborn care, 38% (27/71) of nursing assistants, 45.8% (33/72) of general nurses and 62.5% (25/40) of midwives were judged to have adequate knowledge. The level of knowledge among general nurses was not statistically different compared to nursing assistants (*p* = 0.345). Midwives significantly had adequate knowledge compared to nursing assistants (*p* = 0.014). Only 15.5% (11/71) of nursing assistants, 11.1% (8/72) of general nurses and 12.5% (5/40) of the midwives were considered to have adequate knowledge in managing infections of the newborn. There was no statistical difference in the level of knowledge among general nurses (*p* = 0.442) and midwives (p = 0.667) compared to nursing assistants.

About 38% (27/71) of nursing assistants, 69.4% (50/72) of general nurses and 65% (26/40) of midwives were considered to have adequate knowledge in identifying and caring for LBW babies. Compared to nursing assistants, general nurses (*p* < 0.001) and midwives (p = 0.007) significantly had adequate knowledge in identifying and stabilizing LBW babies (Table 4).

#### Level of care

Nearly equal proportions of health workers 53.2% (59/111) and 54.2% (39/72) deployed at health centre levels III/II and the hospital/health centre level IV respectively, were considered to have adequate knowledge in prenatal care. There was no statistical difference in the level of prenatal knowledge between health workers that were deployed in the hospital/health centre level IV (*p* = 0.893) compared to those deployed at health centres of levels III/II.

About 44.1% (49/111) of health workers deployed at the health centre levels III/ II and 50% (36/72) of those deployed at hospital/ HC IV were considered to have adequate knowledge in immediate newborn care. However their difference in knowledge was not statistically significant (*p* = 0.438). Only 12.6% (14/111) of health workers based at health centre levels III/II and 13.9% (10/72) based at the hospital/health centre of level IV were judged to have adequate knowledge in managing infections of the newborn. There was no statistical difference in knowledge between the two levels of care (*p* = 0.803). With regards to identifying and stabilizing LBW babies, 54.1% (60/111) of health workers deployed at health centre of levels III/II and 59.7% (43/72) deployed at the hospital/health centre level IV were considered to have adequate knowledge. There was no statistical difference in the level of knowledge between the two categories (*p* = 0.450).

#### Years of service

Regarding health worker knowledge about prenatal care, 49.4% (39/79) of health workers who had served for five years or less and 56.7% (59/104) of those who had served six years or longer were considered to have adequate knowledge. There was no difference in prenatal knowledge between health workers who had served six years or more compared with those who had served five years or less (*p* = 0.324).

In terms of immediate newborn care, 45.6% (36/79) and 47.1% (49/104) of health workers who had served six years or more and five years or less respectively were considered to have adequate knowledge. There was no statistical difference in knowledge between health workers who had served six years or longer in reference to health workers who had served five years or less (*p* = 0.836).

Just 10.3% (8/79) and 4.8% (5/104) of health workers who had served for five years or less and six years or longer respectively were judged to have adequate knowledge in managing infections of the newborn. The knowledge difference in managing infections of newborns was not statistically significant (*p* = 0.875). About 55.7% (44/79) of health workers who have served five years or less and 56.7% (59/104) who had served six years or more were judged to have adequate knowledge in identifying and stabilizing LBW babies. But the difference in knowledge was not statistically significant (*p* = 0.889).

## Discussion

In this study we aimed to determine the level of health worker knowledge regarding recommended prenatal and newborn care. Our primary target of health workers were general nurses, midwives and nursing assistants.

### Low level of knowledge

The most striking findings were the general low level of knowledge among health workers regarding prenatal and newborn care whereby fewer than 60% of health workers were considered to have adequate knowledge in prenatal care, immediate newborn care or identifying and stabilizing LBW babies. Knowledge regarding infection management in newborn babies was considered least with only 7.1% of health workers judged to have adequate knowledge. Contrary to our findings, a study conducted in eastern Uganda reported all health workers rated themselves to be competent in providing health care [10], although this particular study suffered from the weakness of self rated questions which are subjective in nature. In Pakistan, though a quite different context from ours, a similar study that examined the knowledge of health workers regarding maternal, child and newborn health found that the level of knowledge was low for all levels of cadres [16]. In their study, the authors suggested periodic training-needs assessment for health workers in order to institute appropriate training interventions. A similar recommendation can be adapted in the case of Masindi district.

Just over half of health workers were considered to have adequate knowledge in prenatal care. This low proportion was attributable to lower proportions of health workers that could correctly state optimal timing (27.9%) and routine prenatal activities (39.9%). Low levels of prenatal knowledge among health workers implies that pregnant women are likely to receive incomplete information and hence leaving them less prepared for their pregnancy, childbirth and newborn care [25].

There were few differences in the levels of knowledge between the different groups of cadres, ranging from relatively higher qualified general nurses and midwives to less qualified nursing assistants. This raises two fundamental concerns: first on the quality of training of the former category [25]; and second, it adds to the debate of delegation of tasks to less qualified staffs [26]. On the one hand, our results demonstrate that delegation is possible [27] given no difference in the level of knowledge between the different categories of health workers. On the other hand, it raises doubts because knowledge was found to be inadequate for all cadres therefore making delegation less desirable. We suggest a similar knowledge assessment for clinical officers and medical doctors that are higher qualified compared to the nursing cadres.

Less than 50% of health workers were judged to have adequate knowledge in immediate newborn care. This means that women in the immediate postnatal period may not receive relevant information about breastfeeding, hygienic cord and thermal care. This may partly explain why many postnatal mothers delay to initiate breastfeeding, apply animal wastes on the umbilical stump, and bath their babies soon after birth [28]. In addition, health workers were not aware of the optimal period for postnatal check-ups for both mother and newborn. The first postnatal check-up is expected to occur within the first three days of birth since this is considered the most dangerous time for newborn babies [29]. In case of illness, newborns are likely to present late to the health facility, usually in critical conditions therefore increasing the chances of dying from hitherto preventable causes.

Less than half of health workers were assessed to have adequate knowledge in managing infection or identifying and stabilizing LBW babies. Infections and LBW babies among newborns are leading causes of morbidity and mortality, and contribute between 56-66% to newborn mortality [12,30,31]. Prompt initiation of therapy is dependent on early detection of infection based on common clinical signs since precise laboratory technologies are often lacking in resource constrained rural areas [3]. Similarly, to initiate appropriate and timely intervention, correct identification of a LBW baby is crucial. LBW increases vulnerability of newborns to hypothermia, infections and poor breastfeeding habits [32,33]. It is likely that delayed detection and inappropriate treatment occur due to low level of knowledge among health workers hence contributing to high morbidity and mortality rates in Masindi district.

### Differences in level of knowledge

Our results showed that general nurses significantly had adequate knowledge in identifying and stabilizing LBW babies. In part, the differences in knowledge can be explained by the differences in pre-service training on prenatal and delivery care that nurses received. Surprising to us, the difference in knowledge levels among midwives compared with nursing assistants was not statistically significant. This could be due to the conservative Bonferroni technique used in data analysis that adjusted our *p*-value to 0.004 from 0.05. We expected to find higher level of knowledge among experienced health workers who had served for six years or higher. Conversely, health workers with fewer years of service should be more knowledgeable since their training/education was more recent than those who have served for longer. These workers should have the more recent/updated recommendations related to prenatal and newborn care. These ambiguous findings may point to the training curriculum for general nurses and midwives or in-service training or supportive supervision aspects not explored in this study.

Health workers deployed to higher levels of care (hospital/health centre level IV) were considered to have similar level of knowledge to health workers deployed to lower levels (health centre level III/II). This finding holds implications for the referral system in Masindi. The referral system is organized such that difficult conditions are referred for better management from lower to higher levels of care as referring implies a significant gradient in knowledge as well as competences and skills. The current situation raises further questions on how supervisory roles of the hospital/health centre level IV to health centre levels III/II can be organised and implemented.

Overall, our data shows that health workers were more knowledgeable in prenatal, immediate newborn care and identifying and stabilizing LBW babies compared to care for infections in newborn babies. Clinical treatment guidelines and “mothers’ passport” have been widely circulated in all health facilities in the country. The ‘mothers’ passport’ outlines preventive interventions while clinical treatment guidelines detail management of infection and LBW babies. A knowledge and decision study conducted in Ghana revealed that these guidelines are seldom used by health workers [34]. It might be that similarly, in Masindi health workers do not make reference to the available materials.

### Study limitations

This study compared knowledge levels between midwives, general nurses and nursing assistants that have different pre-service training backgrounds. However, during practice all health workers are expected to offer standard care to prenatal, postnatal and newborn babies. We did not specifically assess for other health system factors such as supervision, in-service training, the use of guidelines and other materials that could have also influenced the level of knowledge among health workers [35]. Knowledge scores were low partly because of the stiff cut-off points suggested for the category ‘adequate knowledge’. However it was necessary to obtain a clearer picture of the knowledge gap in order to better inform subsequent implementation projects that aim to mitigate them. The Bonferroni adjustment for multiple test is a conservative methodology and could have further under-estimated the level of significance in some cases [23].

## Conclusion

Primary health care workers who make contact with pregnant women and newborn babies in Masindi district have very low level of knowledge regarding prenatal, postnatal and immediate newborn care. Low level of knowledge especially regarding neonatal infection and caring for LBW babies should be considered an important concern for the health system in Masindi, since this category of newborns are also the most at risk. Other health system problems notwithstanding, low level of health worker knowledge regarding prenatal and immediate newborn care presents a major bottle-neck to neonatal mortality reduction in Masindi. This may be a similar problem across Uganda and other sub-Saharan countries.

A deliberate effort should be instituted to update health workers in Masindi on recommended prenatal and newborn care practices such as basic information to be offered to prenatal women. Particular attention should be paid to neonatal infection management.

## Competing interests

The authors declare that there is no competing interests.

## Authors’ contributions

Conceptualized and designed the study RMA, CGO, BC & PK; conducted and entered data RMA; analyzed the data RMA & EN; wrote the manuscript RMA, EN, CGO, BC & PK; BC, CGO and PK provided oversight and needed technical support. All authors read and agreed on the final version.

## Pre-publication history

The pre-publication history for this paper can be accessed here:

http://www.biomedcentral.com/1472-6963/14/65/prepub

## Supplementary Material

Additional file 1 Multiple Choice Questions administered to health workers. Click here for file

## References

[B1] Lawn JE Cousens S Zupan J 4 million neonatal deaths: when? Where? Why? Lancet 2005 14 9462 891 900 10.1016/S0140-6736(05)71048-5 15752534

[B2] UNICEF: Levels and trends in Child Mortality Report 2012 Estimates Developed by the UN Interagency Group for for Child Mortality Estimates (UNICEF/WHO/World Bank/United Nations) 2012 New York USA: United Nations Children’s fund

[B3] Ganatra HA Zaidi AK Neonatal infections in the developing world Semin Perinatol 2010 14 6 416 425 10.1053/j.semperi.2010.09.004 21094416

[B4] Shiffman J Issue attention in global health: the case of newborn survival Lancet 2010 14 2045 2049 10.1016/S0140-6736(10)60710-6 20569844

[B5] Bhutta Z Ali S Samana A Cousens S Ali TM Haider BA Rizvi A Okong P Bhutta SZ Black RE Alma-Ata: rebirth and revision 6: interventions to address maternal, newborn, and child survival: what diff erence can integrated primary health care strategies make? Lancet 2008 14 972 989 10.1016/S0140-6736(08)61407-5 18790320

[B6] Damstadt GL Bhutta ZA Cousens S Taghreed A Neff W de Bernis L Team ftLNSS Evidence-based, cost-effective interventions: how many newborn babies can we save? Lancet 2005 14 977 988 10.1016/S0140-6736(05)71088-6 15767001

[B7] Martines J Paul VK Bhutta ZA Koblinsky M Soucat A Walker N Bahl R Fogstad H Costello A Neonatal survival: a call for action Lancet 2005 14 9465 1189 1197 10.1016/S0140-6736(05)71882-1 15794974

[B8] Health Newborn Networks Innovations in maternal, newborn and child health http://www.healthynewbornnetworkorg/blog/innovations-maternal-newborn-and-child-health. 2013 (accessed 23 August 2013)

[B9] WHO The Global strategy for women’s and children’s health 2010 Geneva: World Health Organization

[B10] Lutwama GW Roos JH Dolamo BL A descriptive study on health workforce performance after decentralisation of health services in Uganda Hum Resour Health 2012 14 1 41 10.1186/1478-4491-10-41 23134673 PMC3506521

[B11] Agrawal PK Agrawal S Ahmed S Darmstadt GL Williams EK Rosen HE Kumar V Kiran U Ahuja RC Srivastava VK Effect of knowledge of community health workers on essential newborn health care: a study from rural India Health Policy Plan 2012 14 2 115 126 10.1093/heapol/czr018 21385799 PMC3606030

[B12] Waiswa P Kallander K Peterson S Tomson G Pariyo GW Using the three delays model to understand why newborn babies die in eastern Uganda Trop Med Int Health 2010 14 8 964 972 10.1111/j.1365-3156.2010.02557.x 20636527

[B13] Rath K Swain BK Mishra S Patasahani T Kerketta AS Babu BV Peripheral health workers’ knowledge and practices related to filarial lymphedema care: a study in an endemic district of Orissa, India Am J Trop Med Hyg 2005 14 4 430 433 15827281

[B14] MoH: Health Sector Strategic and Investment Plan III Promoting People’s Health to Enahnce Socio-Economic Transformation (2010/2011-2014/2015) 2010 Kampala: The Ministry of Health

[B15] Rutebemberwa E Pariyo G Peterson S Tomson G Kallander K Utilization of public or private health care providers by febrile children after user fee removal in Uganda Malar J 2009 14 45 10.1186/1475-2875-8-45 19284673 PMC2657913

[B16] Ariff S Soofi SB Sadiq K Feroze AB Khan S Jafarey SN Ali N Bhutta ZA Evaluation of health workforce competence in maternal and neonatal issues in public health sector of Pakistan: an assessment of their training needs BMC Health Serv Res 2010 14 319 10.1186/1472-6963-10-319 21110888 PMC3012669

[B17] Murila F Obimbo Madadi M Musoke R Assessment of knowledge on neonatal resuscitation amongst health care providers in Kenya Pan Afr Med J 2012 14 78 PMC3361216 22655112

[B18] Monebenimp F Tenefopa M Mve Koh V Kago I Competence of health care providers on care of newborns at birth in a level-1 health facility in Yaounde, Cameroon Pan Afr Med J 2012 14 45 PMC3343673 22593781

[B19] Kish L Survey Sampling 1965 New York: John Wiley & SOns, Inc.

[B20] Eriksson L Nga NT Malqvist M Persson LA Ewald U Wallin L Evidence-based practice in neonatal health: knowledge among primary health care staff in northern Viet Nam Hum Resour Health 2009 14 36 10.1186/1478-4491-7-36 19393073 PMC2678076

[B21] Bhutta ZA Darmstadt GL Hasan BS Haws RA Community-based interventions for improving perinatal and neonatal health outcomes in developing countries: a review of the evidence Pediatrics 2005 14 2 Suppl 519 617 15866863 10.1542/peds.2004-1441

[B22] GoU Service MoP The Uganda Public Service Standing Orders 2010 2010 Kampala: Government of Uganda

[B23] Perneger TV What’s wrong with Bonferroni adjustments BMJ 1998 14 7139 1236 1238 10.1136/bmj.316.7139.1236 9553006 PMC1112991

[B24] Bland JM Altman DG Multiple significance tests: the Bonferroni method BMJ 1995 14 6973 170 10.1136/bmj.310.6973.170 7833759 PMC2548561

[B25] Ayiasi MR Van Royen K Verstraeten R Atuyambe L Criel B Garimoi CO Kolsteren P Exploring the focus of prenatal information offered to pregnant mothers regarding newborn care in rural Uganda BMC Pregnancy Childbirth 2013 14 1 176 10.1186/1471-2393-13-176 24041135 PMC3848633

[B26] Dambisya YM Matinhure S Policy and programmatic implications of task shifting in Uganda: a case study BMC Health Serv Res 2012 14 61 10.1186/1472-6963-12-61 22409869 PMC3352120

[B27] Huicho L Scherpbier RW Nkowane AM Victora CG How much does quality of child care vary between health workers with differing durations of training? An observational multicountry study Lancet 2008 14 9642 910 916 10.1016/S0140-6736(08)61401-4 18790314

[B28] Waiswa P Peterson S Tomson G Pariyo GW Poor newborn care practices - a population based survey in eastern Uganda BMC Pregnancy Childbirth 2010 14 9 10.1186/1471-2393-10-9 20178626 PMC2834614

[B29] WHO/UNICEF Home Visits for the Newborn Child: A Strategy to Improve Survival 2009 Geneva: WHO/UNICEF Joint Statement; World Health Organisation 24809117

[B30] Bhutta ZA Belgaumi A Abdur Rab M Karrar Z Khashaba M Mouane N Child health and survival in the Eastern Mediterranean region BMJ 2006 14 7573 839 842 10.1136/bmj.38979.379641.68 17053238 PMC1618443

[B31] WHO The Global burden of Disease: 2004 updates 2008 Geneva: World Health Organization

[B32] Kumar V Shearer JC Kumar A Darmstadt GL Neonatal hypothermia in low resource settings: a review J Perinatol 2009 14 6 401 412 10.1038/jp.2008.233 19158799

[B33] Knobel RB Holditch-Davis D Schwartz TA Wimmer JE Jr Extremely low birth weight preterm infants lack vasomotor response in relationship to cold body temperatures at birth J Perinatol 2009 14 12 814 821 10.1038/jp.2009.99 19626030 PMC2787712

[B34] Oduro-Mensah E Kwamie A Antwi E Amissah Bamfo S Bainson HM Marfo B Coleman MA Grobbee DE Agyepong IA Care decision making of frontline providers of maternal and newborn health services in the greater accra region of Ghana PLoS One 2013 14 2 e55610 10.1371/journal.pone.0055610 23418446 PMC3572062

[B35] Sipsma HL Curry LA Kakoma JB Linnander EL Bradley EH Identifying characteristics associated with performing recommended practices in maternal and newborn care among health facilities in Rwanda: a cross-sectional study Hum Resour Health 2012 14 1 13 10.1186/1478-4491-10-13 22776289 PMC3444308

